# Structure and function of capsid protein in flavivirus infection and its applications in the development of vaccines and therapeutics

**DOI:** 10.1186/s13567-021-00966-2

**Published:** 2021-06-30

**Authors:** Xingcui Zhang, Yanting Zhang, Renyong Jia, Mingshu Wang, Zhongqiong Yin, Anchun Cheng

**Affiliations:** 1 Research Center of Avian Disease, College of Veterinary Medicine, Sichuan Agricultural University, Chengdu, 611130 Sichuan China; 2 Institute of Preventive Veterinary Medicine, College of Veterinary Medicine, Sichuan Agricultural University, Chengdu, 611130 Sichuan China; 3 Key Laboratory of Animal Disease and Human Health of Sichuan Province, Chengdu, 611130 Sichuan China

**Keywords:** Flavivirus, capsid protein, ecapsidation, vaccine, therapeutic

## Abstract

Flaviviruses are enveloped single positive-stranded RNA viruses. The capsid (C), a structural protein of flavivirus, is dimeric and alpha-helical, with several special structural and functional features. The functions of the C protein go far beyond a structural role in virions. It is not only responsible for encapsidation to protect the viral RNA but also able to interact with various host proteins to promote virus proliferation. Therefore, the C protein plays an important role in infected host cells and the viral life cycle. Flaviviruses have been shown to affect the health of humans and animals. Thus, there is an urgent need to effectively control flavivirus infections. The structure of the flavivirus virion has been determined, but there is relatively little information about the function of the C protein. Hence, a greater understanding of the role of the C protein in viral infections will help to discover novel antiviral strategies and provide a promising starting point for the further development of flavivirus vaccines or therapeutics.

## Introduction

*Flavivirus* is part of the *Flaviviridae* family along with *Pestivirus*, *Hepacivirus* and *Pegivirus* [1, 2]. The primary categories are mosquito-borne (such as Usutu virus, USUV; dengue virus, DENV; Japanese encephalitis virus, JEV; West Nile virus, WNV; Zika virus, ZIKV) and tick-borne flaviviruses (such as tick-borne encephalitis virus, TBEV). These flaviviruses mainly cause foetal malformation (such as ZIKV) and neurological disorders (such as JEV and TBEV). A flavivirus is an enveloped single positive-strand RNA virus with a genome of approximately 11 kb that contains a single open reading frame (ORF) flanked with a short (~100 nt) 5′-untranslated region (UTR) and a longer (~400–600 nt) 3′-UTR [3]. The ORF encodes a polyprotein associated with the endoplasmic reticulum (ER) membrane and is proteolytically cleaved into three structural proteins (capsid, C; precursor membrane, prM; and envelope, E) and at least seven nonstructural (NS) proteins (NS1, NS2A/B, NS3, NS4A/B and NS5) by host and viral proteases [4]. The C protein is essential for the assembly and maturation of viral particles and is the most promising drug target candidate. Before being cleaved by a viral protease to produce a mature protein, the C protein takes the form of a membrane-anchored C (anchC), which can initiate subsequent effective flavivirus assembly but will not perform viral replication [5], and this process induces the downstream prM to be cleaved into Pr and M by furin [6]. anchC is also the signal peptide that transfers prM to the ER lumen. Therefore, anchC is produced and disappears in the life cycle of flavivirus, and its mechanism of action in virus assembly may be helpful for antiviral research. However, the size and transient existence of anchC may limit its research. The nonstructural proteins are proteases that are mainly involved in the cleavage of polyprotein [7] and the regulation of host cell responses [8]. NS2A and NS3 also participate in the assembly of virions through direct interaction with the C protein [9]. NS1 regulates the production of infectious particles by interacting with structural proteins (C, prM and E) [10, 11].

**Figure 1:**
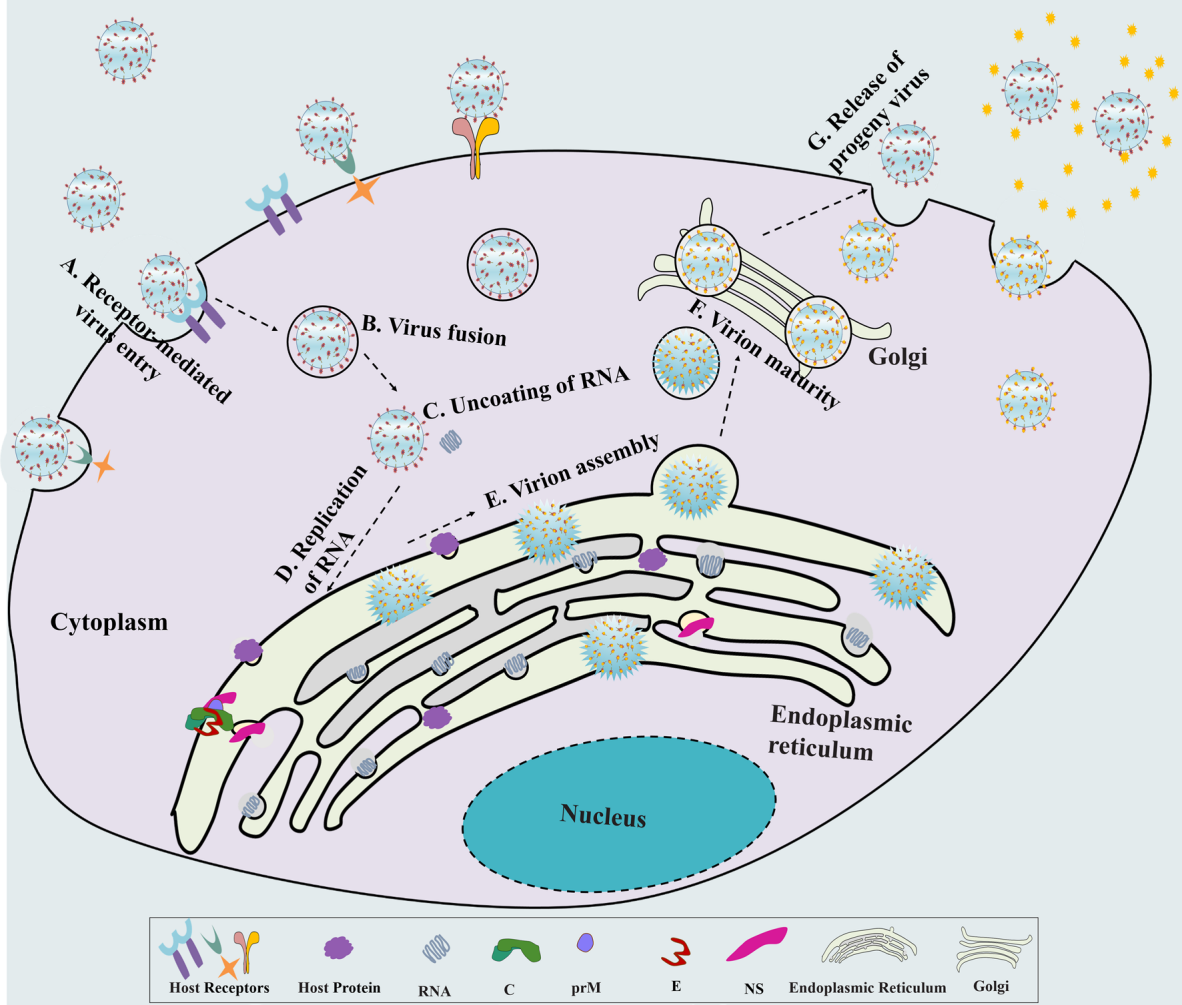
**Propagation process of flaviviruses.** Flavivirus propagation mainly includes adsorption, entrance, replication, assembly, maturity and release. The virus enters host cells through host receptor recognition (A); then, the virus fuses with the host membrane (B), and the RNA of flavivirus is ejected from the nucleocapsid and released into the cytoplasm of the host cells (C). RNA replication, protein synthesis and particle assembly are carried out in the ER (D and E); the particles mature in the Golgi apparatus (F); subsequently, mature virions are released from the host cells (G).

Flaviviruses use a complex reproduction process to gain access to host cells (Figure 1). Flavivirus infection can induce invagination of the ER, forming clusters of double-membrane vesicles (Ves) wrapped in vesicle packets (VPs) [12]. The Ve houses the viral replication complex, including double-stranded viral RNA, viral nonstructural proteins and cellular proteins [13]. C protein binds to the membrane and exits the ER via a coat protein complex II (COPII)-dependent mechanism, bypassing the Golgi apparatus; the presence of interferon-induced protein viperin enhances the release of C protein [14]. Currently, there is relatively little information on the functional properties of flavivirus C protein. However, C protein is the critical element of infectious virus particles and contains positively charged residues distributed throughout the molecule. The mature C protein assembles on the genomic RNA through nonspecific electrostatic interactions to form a nucleocapsid, which is wrapped by the lipid bilayer with prM and E proteins to form an infectious virion [15, 16]. The purified C protein dimer can be assembled into C-like particles when combined with transcribed viral RNA in vitro [17], and the C protein-coding region hairpin sequence (cHP) is a conserved region in arthropod-borne flaviviruses. cHP is involved in the enhanced recognition of translation initiation codons, which are essential for effective RNA replication [18]. In this review, we primarily summarize the structure and function of the C protein, aiming to discover more new functions and assist in the development of flavivirus vaccines and drugs.

## Structure of the C protein

The structure of the C protein has been solved by nuclear magnetic resonance (NMR) and X-ray crystallography [19]. The C protein is located at the N-terminus of the polyprotein [20]; it is an internal protein in virus structure, and we expect it to consist of approximately 180 capsid protein units [21]. The C protein is composed of approximately 100 amino acid residues and has a molecular weight of approximately 13 kDa; for example, the C protein of Tembusu virus (TMUV) consists of 120 amino acids (aa), that of WNV contains 105 aa, and that of DENV contains 100 aa. Compared with the other two surface proteins prM and E in different flaviviruses, the C protein has the lowest amino acid homology. The homology of the JEV C protein to WNV, DENV-2 and TBEV is only 67%, 33%, and 25%, respectively. However, certain characteristics, such as the biochemical properties, structural specifics (hydrophobicity, secondary/tertiary structure, and abundance of basic amino acid residues) [22] and several functional elements of the C protein, seem to be well conserved in flaviviruses [23, 24]. These conservative biochemical characteristics contribute to similar functions.

### Superhelical structure of the C protein

**Figure 2:**
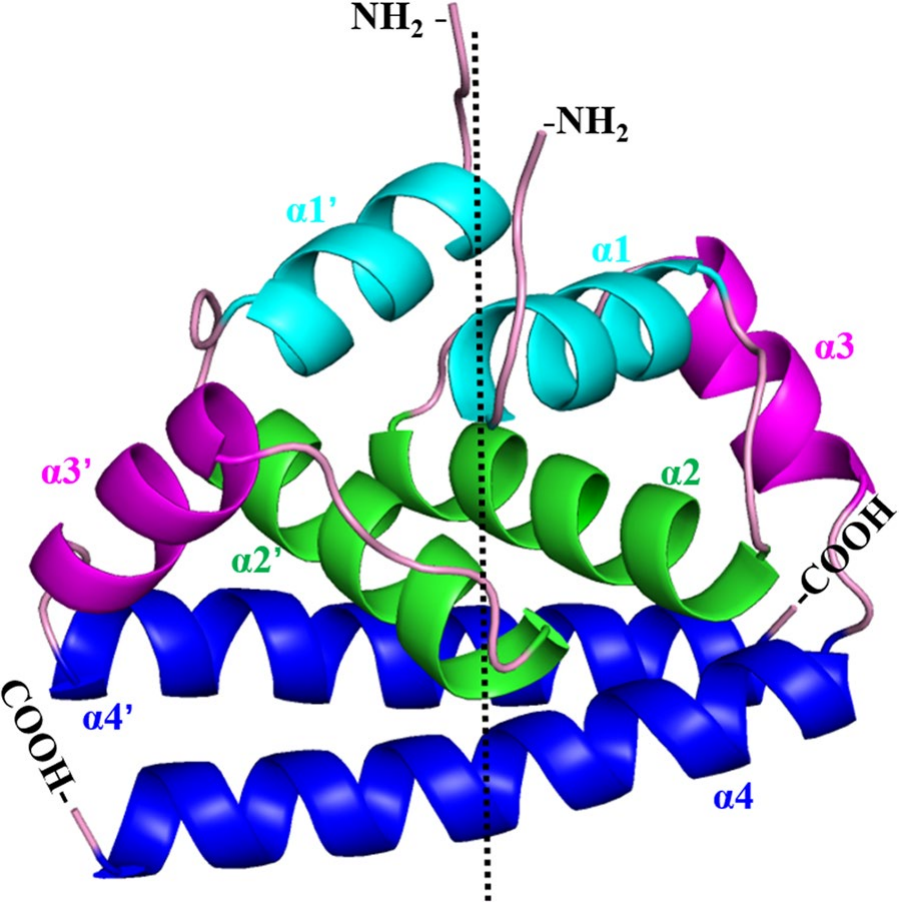
**3D structure of the flavivirus capsid protein.** Topological diagram of a C protein homodimer: α-helices 1–4 of each C monomer are shown in light blue, green, purple and dark blue, respectively. The α1–α1′ and α2–α2′ helices are located on the opposite side of the α4–α4′ helices, and the α3–α3′ helix is parallel to the α4–α4′ helix. The 3D structure was created with PyMOL software.

Each C protein molecule contains two highly conserved internal regions: a hydrophobic region and a highly cationic region [25]. A far-ultraviolet circular dichroism (CD) potassium analysis showed that the flavivirus C protein monomer forms oligomers in solution and that the protein is predominantly a dimer with a two–alpha helical conformation, including four α-helices (α1 to α4) connected by short loops [26] (Figure 2). The C protein dimer is essential for viral assembly and virus particle stability [27]. The α4 helix at the hydrophobic C-terminus of the C protein is the longest of the four helices, and the α4–α4′ interaction plays the most important role in supporting C protein dimers [28], nucleocapsid formation and virus production [29]. The α1–α1′ and α2–α2′ helices are located on the opposite sides of the α4-α4′ helix and are composed of nonpolar residues [30]. Some researchers think that the α4–α4′ region interacts with RNA, and the hydrophobic core in the α2–α2′ region binds to the virus and host lipid membranes [31]. The α1 helix is a part of the central hydrophobic region, which may play an important role in virus assembly and interactions with virus surface proteins [32, 33]. The α3–α3′ helix is parallel to the α4–α4′ helix, and the α1 and α3 helices are amphipathic and are mainly composed of leucine residues.

### Nucleocapsid

**Figure 3:**
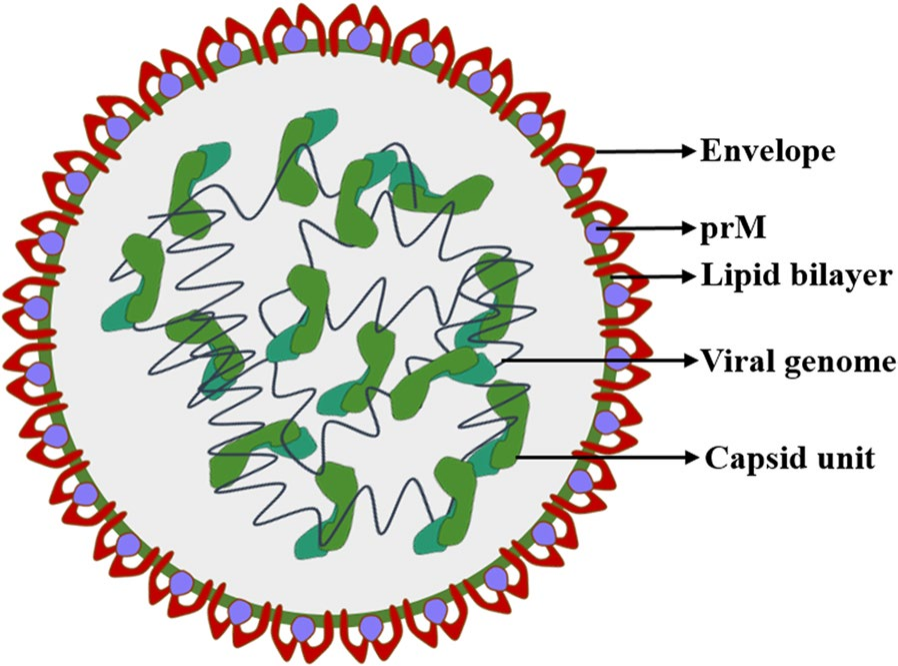
**Infectious particle of the flavivirus.** The viral RNA genome is packaged in a spherical nucleocapsid composed of multiple copies of the C protein. The structural proteins prM/E and lipid bilayer enclose the nucleocapsid core.

The flavivirus particle consists of an electron-dense inner core called the nucleocapsid (NC). The NC complex contains multiple copies of the C protein and a single copy of genomic RNA, and the production of infectious virus particles requires the NC. The anchC may help initiate the formation of the nucleocapsid, including interactions with the genomic RNA. The mature C protein is responsible for packaging the viral nucleic acid and ribonucleoprotein complex in the virion, and RNA encapsidation is the first step in the assembly process of flaviviruses [34]. In the process of encapsidation, the C protein acts as a chaperone of RNA, promoting the folding of RNA by preventing its misfolding or by dissolving misfolded RNA without consuming ATP. The C protein is also involved in regulating the cyclization of flavivirus genome RNA for viral replication [35]. The NC is enveloped by two other structural proteins (prM/E) and the lipid bilayer derived from the host cell ER [36–38] (Figure 3). The interaction between the C protein and nucleolin in the nucleolus is important for the formation of stable and functional nucleocapsids [39]. During the virus infection cycle, the dissociation mode of the NC in the cytoplasm is not yet completely understood and may be worth exploring in antiviral studies.

### Nuclear localization signals

The C protein of flavivirus is localized in both the cytoplasm and the nucleus. The C protein has basic amino acid regions (mainly 23–25% Arg and Lys) called nuclear localization signals (NLSs), and it can interact with nuclear import receptor proteins (such as transcription factor hnRNP K, nucleophosmin and importin α/β), which promote entry of the C protein into the nucleus; the NLSs of WNV are located from aa 85 to aa 101 [40], and the NLSs of DENV are located from aa 85–100, including ^6^KKAR^9^, ^73^KKSK^76^, and the bipartite signal ^85^RKeigrmlnilnRRRR^100^ [41–43]. The active transport system may be related to the movement of C protein from the cytoplasm to the nucleus, and importins (α/β), protein kinase and C protein-mediated phosphorylation are involved in this process [40]. The interaction between the C protein and importin-α is mediated by NLS motifs, but the function of the C protein in the nucleolus is almost unknown. According to evolutionary study of the *Flaviviridae*, C protein NLSs are highly conserved in mosquito-borne flavivirus and blood-borne and human-adapted hepatitis C virus (HCV). The Gly^42^ and Pro^43^ of C proteins are important for nuclear localization and are completely conserved in flavivirus and HCV [44]. After mutating Gly^42^ and Pro^43^ to Ala (the M4243 mutant), an increase in defective particles or a low viral RNA replication efficiency was detected in JEV [45].

### Phosphorylation sites

The NetPhos algorithm shows that flavivirus C protein has 2–6 putative phosphorylation sites. These phosphorylation sites exist in the RNA-binding regions of the C protein. Therefore, dephosphorylation of the C protein enhances its interaction with viral RNA. According to bioinformatics analyses, the WNV C protein is phosphorylated in infected cells and has 5 putative phosphorylation sites (serine 26, 36, 83, 99 and threonine 100). The work of Cheong [46] showed that mutation in these phosphorylation sites of the C protein reduced its RNA binding activity and did not inhibit oligomerization but did affect the ratio of dimerization and oligomerization. In infected cells, the flavivirus C protein is localized in the nucleus due to the presence of NLSs. Interestingly, the degree of phosphorylation of the C protein is reduced, thereby reducing its nuclear localization [47].

### Structural flexibility of the C protein

The N-terminal region of the C protein is unstructured in solution, especially the first 20 residues [48]. This region has abundant positive charges because it is rich in the basic residues Arg and Lys. Therefore, almost 40% of the unstructured N-terminus of the C protein can be removed without severely damaging its functional integrity. Hence, truncation of the N-terminal region has been used in the prokaryotic expression and purification of recombinant C. The truncated C protein retains the ability to package RNA, and even the version with approximately 30% of the C-terminal region deleted (including complete deletion of α4) can still function [27]. Removing the entire internal hydrophobic domain from the α2 helix and the partial loop between the α2 and α3 helices of the WNV C protein severely impairs viral growth; however, a short deletion did not substantially affect the growth, indicating the structural and functional flexibility of the C protein [44, 49]. A TBEV study showed that the C-terminal region of the C protein has two amino acid sequence motifs that match the canonical NS2B/NS3 recognition site, and this region has significant functional flexibility in the assembly of infectious virions [50]. The flexibility of the C protein is conferred by its inherent disordered region and is conserved in all flavivirus C proteins. The key to the many actions that C proteins can perform in the proliferation cycle of virus particles and cells is the existence of internal disordered domains.

### *Cis*-acting elements

*Cis*-acting elements are necessary for genome circularization and viral enzyme activity in replication, and the rate of viral replication is very sensitive to small changes in this RNA. The N-terminus of the C protein-coding region is important in the translation, replication, host adaptation and encapsidation of mosquito-borne flavivirus [29, 51]. A novel *cis*-acting element downstream of the 5′ cyclization sequence pseudoknot (DCS-PK) is conserved in mosquito-borne flaviviruses [52], which mainly enhances flavivirus RNA replication by regulating genome cyclization. The function of DCS-PK depends mainly on its secondary structure and some conserved primary sequences, such as the highly conserved stem 1 loop 2 sequence [53]. Stem-loop 6 (SL6) is a *cis*-acting enhancer in the C protein coding region of tick-borne flavivirus [54]. *Cis*-acting elements can be used to study the function of flavivirus C proteins.

## Multiple functions of the C protein

**Figure 4:**
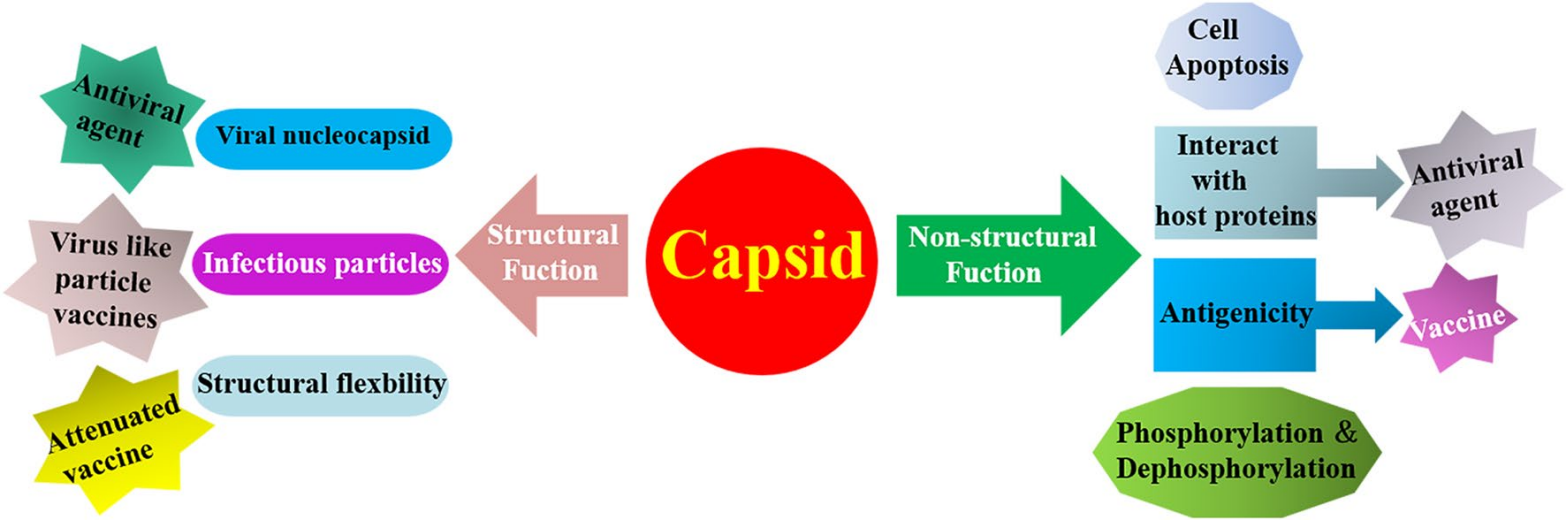
**Multiple functions of the C protein.** The C protein is a flavivirus structural protein that has both structural and nonstructural functions. Vaccines and antiviral drugs can exploit various functional characteristics of the C protein.

The multiple functions of the flavivirus C protein are attributed to the abovementioned special structural features (Figure 4). The C protein undergoes various conformational changes and participates in the formation of the nucleocapsid to protect viral RNA. It also has nonstructural functions in the virus life cycle, mainly involving intermolecular interactions, such as interaction with organelle membranes to promote virus replication, assembly and virion maturation.

### Binding and interacting with RNA

The NS2A protein of flavivirus can recruit genomic RNA, structural proteins (C/prM/E), and nonstructural proteins (NS2B/NS3) to the virion assembly site. Once the C-prM-E polyprotein is formed, NS2A will transfer the viral RNA to the structural proteins for virion assembly [36]. The C protein binds and interacts with RNA to form a nucleocapsid and is wrapped by prM/E proteins. The mutation of NS2A weakens the binding and eliminates virus production, which indicates that NS2A plays an indispensable role in virion assembly [55].

Cell-penetrating peptides (CPPs) can translocate to the cell membrane and function to reduce immunogenicity and cytotoxicity. Recent publications indicate that CPPs can deliver many bioactive molecules such as proteins, nucleic acids, and therapeutics in mammalian cells [56]. CPPs derived from the DENV C protein are involved in suitable viral delivery systems, which can deliver nucleic acids to cells for virus infection or deliver therapeutic molecules [21, 57]. CPPs contain 6–30 amino acid residues, which range from highly charged (rich in Arg and Lys) to hydrophobic or amphipathic sequences. Basic residues are cumulatively distributed on the surface of the C monomer; the positive charges of these basic residues may play a role in the neutralization of negative charges during viral RNA encapsidation. To assemble the nucleocapsid and package the genome without nucleases, the hydrophobic amino acids at the carboxyl terminus of the C protein are fixed to the rough ER of the host cell.

The structural part of the DENV C protein includes two domains, pepR and pepM, which have very distinct physical and chemical characteristics [21]. They are two novel intrinsic CPPs that have conserved folds in the C proteins of the *Flaviviridae* family [58], and they have two different internalization routes. pepR uses the endocytic pathway, while pepM directly translocates physically through the lipid membrane. These two peptides preferentially bind to anionic lipid membranes in an α-helical configuration [21]. pepM is a relatively hydrophobic domain, while pepR is a highly cationic helix. Therefore, pepM primarily interacts with lipid membranes, and pepR binds to RNA in the virion. However, only pepM can promote the fusion and aggregation of Ve, which is composed of zwitter-ionic lipids [59]. The C protein of yellow fever virus (YFV) has a high-affinity dsRNA-binding function, which can interfere with the cleavage of long dsRNA by Dicer, thereby antagonizing RNA silencing [60]. The conservation of the viral suppressor function in various flavivirus C proteins is particularly fascinating. The basic residues in the N-terminal region help RNA bind to form particles [61]. Viral protein-derived CPPs can be a valuable tool for drug delivery across membranes, mainly in genetic therapy. Therefore, researchers designed and synthesized pepM and pepR based on the two domains of the C protein sequence. They have almost equivalent efficiency to the whole parental C protein for ssDNA delivery. In addition to the CPP properties, pepR also has antibacterial activity.

Based on the combination of C protein and RNA, researchers have considered fusing exogenous genes with C protein through a capsid-targeted antiviral inactivation (CTVI) strategy [62]. This tactic is based on the fusion of the C protein and a crucial effector molecule, such as a nuclease, a lipase, a protease, or a single-chain antibody (scAb), which can degrade viral DNA/RNA or interfere with the proper folding of important viral proteins [63].

### Promoting the proper assembly of infectious particles

The C-terminus of the C protein contains a single transmembrane domain (TMD) called anchC, which acts as a signal for the translocation of prM into the ER lumen and controls the stability of the E protein, so it is available for the assembly of infectious particles [64, 65]. The TMD is cleaved by the viral protease NS2B/NS3 to release a functional cytosolic form. In addition, due to the internal hydrophobic sequence (IHS), mature C protein can bind to cellular and intracellular membranes. IHS contains 14 to 22 hydrophobic residues, which are conserved in all flavivirus C proteins and are required for virus maturation and assembly [27]. The C and prM proteins are connected by the IHS, which crosses the ER membrane and is responsible for the translocation of prM to the ER lumen [50, 66]. In a TBEV study, Kofler interfered with the assembly of viral particles by deleting HIS [32], and the proportion of subviral particles (SVPs, lacking the nucleocapsid and infectivity) produced by deletion mutants increased. However, SVPs are highly immunogenic because of the prM and E proteins [67]. Moreover, the C protein plays a vital role in other aspects of the flavivirus replication cycle. Kofler et al. [32] proved that large deletions in the C protein (C △28–35, C △28–39, C △28–43, and C △28–48) did not significantly reduce the level of the C protein in BHK-21, and C protein deletion mutants did not impair RNA replication or translation. However, these mutations can affect the expression of the E protein, which indirectly affects the formation of particles. For example, the diameter of capsid-free and noninfectious SVPs is smaller than that of wild-type viral particles, the viral infectious titres of the four mutants are lower than that of wild-type virus, and the production of infectious particles released by all mutants is lower than that of wild-type virus.

### Phosphorylation and dephosphorylation of the C protein affect virus replication

The phosphorylation of the C protein is an essential process of virus replication in many viruses. It is an important posttranscriptional modification required for flavivirus C protein function, such as binding to importin-α or HDM2 protein [47]. Experiments with kinase inhibitors and activators have shown that protein kinase C is responsible for the phosphorylation of the C protein [47]. C protein undergoes spontaneous self-oligomerization, which is involved in nucleocapsid formation; however, phosphorylation reduces both the oligomerization rate and phosphorylation-dependent nucleocapsid assembly [46]. Dephosphorylation of the C protein is also critical for reducing its nuclear localization. Therefore, during viral infection, the phosphorylation of the C protein should decrease over time. The assembly of flavivirus occurs in the cytoplasm. In the later stages of infection, dephosphorylation promotes the interaction of C protein with RNA in the cytoplasm. Tracking the cellular location of the C protein during infection indicated that it was localized in the nucleus in the early stages of infection and in the cytoplasm in the late phase of infection. These changes are related to the gradual dephosphorylation of C protein in infected cells. However, in *Flaviviridae*, only the C proteins of HCV and WNV have been shown to be phosphorylated. In fact, the bioinformatics analysis of other flavivirus C proteins revealed multiple putative phosphorylation sites. Therefore, the C proteins of other flaviviruses may also be phosphorylated.

### Interactions with host proteins to promote virus propagation

The C protein binds to viral RNA and has other cellular regulatory functions in infected cells. This protein can mediate host protein expression or interfere with immune recognition [68]. It is also associated with various cellular proteins that contribute to viral pathogenesis [69].

#### Interaction with phospholipid-binding proteins to enhance viral replication

During flavivirus infection, C protein progressively accumulates around lipid droplets (LDs) through a noncanonical function of the COPI system, which provides new ideas for antiviral strategies [70–72]. A disordered N-terminal arm of the C protein is involved in specific interactions with host lipid systems, such as LDs and very low-density lipoproteins (VLDL) [73, 74], implicated in viral assembly, maturation and release. The interactions depend on a high concentration of intracellular potassium ions (K^+^) and are mediated by the surface lipoproteins perilipin 3 α-helix 5 (PLIN3α5) and apolipoprotein Eα-helix 4 (APOEα4). These two proteins are the main ligands of the C protein on the surface of LDs and VLDL [28, 75]. Interestingly, the N-terminal region (first 220 residues) of APOE has multiple motifs that match the C-terminal region (last 220 residues) of PLIN3, and the conserved regions may be primarily involved in specific C protein-LD/VLDL interactions [76]; the L50 and L54 amino acids in the α2 helix of the C protein are crucial for these interactions, which are essential for the viral replication cycle [77]. In the study on the DENV C protein, the authors proved that mutations of these two leucine residues would weaken the interaction between the C protein and LDs. Drugs that disrupt LD biogenesis significantly inhibit viral production during the particle assembly step of the viral replication cycle [78, 79]. Interestingly, the binding of DENV C protein to LDs indicates that a 10 amino acid residue peptide, pep14-23 (^14^NMLKR^18^, similar to the importin α self-inhibitory sequence), may be functionally related to the interaction between the C protein and LDs, which may require an α-helical conformation of the C protein. This sequence is a common motif in mosquito-borne flavivirus C proteins. A peptide (pep14-23) designed using the intrinsically disordered N-terminal region (residues 1 to 26) to inhibit the interaction between the DENV C protein and host LDs [78]. In a study on WNV C protein [80], a similar result was observed: pep 14–23 is a potential inhibitor of C protein binding to the host lipid systems, which may be similar to other flaviviruses. The interaction between the C protein and LDs/VLDL is a key step for the viral replication of flaviviruses; therefore, this may provide a novel antiviral strategy.

#### Interaction with nucleolar proteins to promote nuclear localization

The C protein is transported from the cytoplasm to the nucleus through nuclear pore complexes, which penetrate the double layer of the nuclear envelope. During virus replication, the C protein is located in the nucleus because it interacts with importin in infected cells; however, flavivirus assembly occurs in the cytoplasm [47]. The interaction between the C protein and importin is enhanced by phosphorylation. Hence, dephosphorylation disrupts the interaction, resulting in a decrease in the nuclear localization of the C protein, which provides an opportunity for the assembly of virus nucleocapsid in the cytoplasm. A JEV study [81] demonstrated that the amino acid residues Gly^42^ and Pro^43^ are involved in the binding of the C protein to nucleolar phosphoprotein B23, and the binding site is located at amino acid residues 38–77; however, this interaction is not detected in DENV [82]. Interestingly, the mutation of amino acid residues Gly^42^ and Pro^43^ of the JEV C protein to Ala influences the nuclear localization of the C protein, which leads to impaired virus replication and pathogenicity [83]. Gly^42^ and Pro^43^ are the crucial sites for nuclear localization and are associated with RNA replication, protein synthesis and even propagation in Vero cells [81]. Mori et al. [45] demonstrated that the C protein is present in the nucleolus and cytoplasm of mammalian and insect cell lines after JEV infection or transfection with recombinant plasmids that cause C protein expression. However, the nuclear localization of the JEV C protein has been shown to enhance virus replication.

#### Interaction with nonsense-mediated mRNA decay (NMD) pathway factors

Zika virus (ZIKV) is an emerging mosquito-borne flavivirus related to DENV and WNV. It can infect human cells in vitro, such as neural progenitor cells (NPCs), and disrupt the nonsense-mediated mRNA decay (NMD) pathway. NMD is a cellular mRNA monitoring mechanism required for the development of normal brain size in mice. Krystal et al. showed that cellular NMD factors, such as the central NMD regulator up-frameshift protein 1 (UPF1), can interact with viral C protein. The expression of C protein post-transcriptionally downregulates the level of UPF1 protein. Cellular fractionation studies have shown that the ZIKV C protein specifically targets nuclear UPF1 and can be degraded by the proteasome [84]. ZIKV uses C protein to reduce UPF1 levels and inhibit the antiviral activity of NMD, which in turn contributes to the development of neuropathology in vivo. Similar phenomena were also found in WNV and DENV. Li et al. also found that the host exon-junction complex (EJC) recycling factor PYM1 can interact with C, thereby interfering with the function and location of the EJC protein. However, EJCs have a role in NMD, and they have antiviral effects in DENV, WNV, and ZIKV by indirectly or directly targeting viral RNA. The EJC protein R8M8A can directly bind to WNV RNA, but depletion of PYM1 attenuates the binding of R8M8A to viral RNA, so WNV infection can segregate PYM1 to protect the RNA from decay [23]. In contrast, PYM1, a capsid-interacting protein, plays a pivotal role in HCV infection [85].

#### Interaction with hSes3p protein

In the cytoplasm, human Sec 3 (hSec3p) and Jab 1 proteins can interact with the C proteins of DENV and WNV. Confocal experiments showed that hSec3p and flavivirus C protein colocalized in the cytoplasm and perinuclear regions. hSec3p is an extracapsular complex component, and its main function involves secretory pathways and exocytosis. hSec3p has been shown to be a novel chaperone of WNV and DENV C proteins through the proteasome pathway [8]. The SH2 domain-binding motif (last 15 aa) of hSec3p binds to the first 15 amino acids of C; the amino acid residues in positions 14 (WNV) and 13 (DENV) of the C protein are particularly important for this interaction. The protective effect of hSec3p can affect the transcription and translation of viral RNA by chelating elongation factor 1α (EF1α), thereby regulating virus production [25], which can delay flavivirus infection.

#### Interaction with organelle membranes

C protein is expressed in the ER as a part of the flavivirus polyprotein [86]. A study by Markoff et al. [87] showed that the mature C protein is associated with the ER membrane through the internal hydrophobic region located at the α2/α2’ interface, which is known to be conserved among mosquito- and tick-borne flaviviruses [77, 88, 89]. In the DENV study, the C protein fragment peptide C (pepC) was able to bind negatively charged phospholipid membranes through a charge anchor formed by three positively charged amino acid residues (including Arg-2, Lys-6 and Arg-16) [90]. It interacts with organelle membranes to promote viral replication, virion assembly and viral production. This should be considered in examining the flavivirus life cycle.

#### Interaction with caprin-1

The expression of the C protein plays an important role in regulating the activity, expression or localization of host molecules [91]. In JEV, YFV and ZIKV, C has been shown to interact with its binding partner caprin-1 to inhibit the formation of stress granules (SGs). The Lys^97^ and Arg^98^ amino acid residues in the C protein are important for the interaction with caprin-1, and the mutation of these two amino acids to Ala inhibits the formation of SG and damage virus propagation [92]. However, C protein-mediated suppression of SG formation has not been detected in all flaviviruses; for example, the expression of DENV and WNV C proteins does not significantly block the formation of SG [93]. However, it has been reported that DENV and WNV C proteins interact with other host proteins [94, 95].

### Participation in apoptosis

Another nonstructural function of the multifunctional C protein is involved in apoptosis. The phosphorylation of WNV C protein is very important for its interaction with importin and affects its RNA-binding activity, oligomerization, nuclear localization and apoptosis [46, 96]. In several flaviviruses, C proteins have been shown to be pro-apoptotic. However, in a study by Matt D. Urbanowski, WNV C protein was shown to completely block the apoptosis of infected cells during virus replication. The protective effect is mediated by a phosphatidylinositol 3-kinase (PI3K)-dependent pathway [97]. In the nucleus, nucleolin, the apoptotic protein Daxx [98], core histones (H2A, H2B, H3 and H4), hnRNP-K and Hdm2 (in the case of WNV) interact with the C protein. These interactions affect the induction of apoptosis in the host [96] and regulate transcription, leading to the development of disease. The C proteins of various flaviviruses have pro-apoptotic or anti-apoptotic functions [99, 100].

## Application of the C protein

Flavivirus C protein is indispensable in virus replication and assembly, can undergo immune escape mutations to efficiently avoid the immune system, and it is an important target of T cells during natural infection [101, 102]. C protein has a specific structure, various functions and biological characteristics, which make it a potentially promising drug target for antiviral agents. In a study of DENV, a novel low-molecular-weight compound, ST-148, was used to interact with the C protein and block its activity, which is required for virus replication [103]. The pep14-23 region of the C protein is the major participant in the interaction with LDs. Therefore, a peptide was designed to bind to LDs and inhibit C protein-LD interactions to affect virus replication. The pep14-23 region is also very important for drug design, but further research is needed. Flavivirus C protein has obvious structural functions in mature virions, especially in the processes of viral encapsidation, because it belongs to the same class of α-helical C proteins as hepadnaviruses and retroviruses. Some studies have successfully used flavivirus C protein in the CTVI strategy against DENV [104, 105] and JEV [24]. In addition to flavivirus, the C protein of classical swine fever virus (CSFV) [24, 106] belonging to the *Pestivirus* genus has also been used in CTVI. Based on these antiviral mechanisms, CTVI is expected to be applied to more *Flaviviridae* viruses for targeted antiviral research, such as TMUV, YFV, and the *Pegivirus* genus. However, there are still some problems to be solved if this strategy is applied to clinical research, such as finding more nucleases that are suitable for CTVI and can be tolerated by host cells without cytotoxicity.

Because envelope domain III (EDIII) contains important linear antigenic epitopes that directly interact with neutralizing antibodies [107] and the C protein is important in the flavivirus life cycle, researchers have fused EDIII of the DENV E protein with the C protein of DENV, leading to the formation of the tetravalent vaccine DIIIC [88, 108, 109]. The results demonstrated that DIIIC can induce cell-mediated immunity and has the ability to protect mice against DENV. The structural flexibility of the C protein has been proven in numerous studies. Schlick et al. [110] used a deletion mutant of the C internal hydrophobic domain and another deletion mutation in the 3′ noncoding region (3′-NCR), which removed a conserved hairpin structure (hairpin II-1 nt 10737–10825) to study attenuated vaccine candidates in WNV in vivo. The vaccine is not pathogenic but can induce protective immunity in mice.

Among all flavivirus proteins, the C protein has the lowest conservation. However, the structural properties of the protein are very similar, and its charge distribution is well conserved, which is conducive to the conservation of its function. Table 1 summarizes the numerous functional applications of flavivirus C proteins.

**Table 1:** **Applications of flavivirus capsid proteins**

Proteins	Viruses	Virus strains	Application types	References
EDIII and C	DENV	Hawaii (DENV-1) New Guinea C (DENV-2) H-87 (DENV-3) H241 (DENV-4) DENV-2 A15 DENV-2 SB8553/S16803	Subunit vaccine	[123, 124]
		DENV-1 Jamaica (AF42564) DENV-2 SB8553 DENV-3 (FJ882576) DENV-4 (AF326573)	Vaccine	[125]
		DENV-2 A-15 strain DENV-2 SB8553 DENV-2 Jamaica	Vaccine	[126]
C		Hawaii (DENV-1) New Guinea C (DENV-2) H-87 (DENV-3) H241 (DENV-4) DENV-2 SB8553	Vaccine	[127]
		Four serotypes	Antiviral agent	[128]
C	JEV	Wild-type and 9798A mutant of JEV AT31	Antiviral agent	[92]
		Wild-type and L17A/CSmt JEVs	Pathogenesis of JEV infection	[117]
C	WNV	WNV NY99	Live vaccine candidates	[110]
C	TMUV	DTMUV WR strain	DNA vaccine	[129]
		CQW1 strain	Target therapeutic	[130]
C-prM-E	ZIKV	PRVABC59	Virus-like particle vaccines	[131]
C		FSS13025 strain	Live-attenuated vaccine	[132]
VSV-C		PRVABC59 strain	Vesicular stomatitis virus (VSV)-based vaccine	[102]

## Conclusions

Flavivirus C protein is a multifunctional protein that participates in many aspects of the virus life cycle. The C protein binds to viral RNA to form the nucleocapsid and plays an important role in the process of viral infection, including interactions with cellular proteins and the regulation of cell metabolism, apoptosis and immune responses [111, 112].

As a structural protein, the main function of C involves packaging viral genomic RNA and the formation of the viral core, while anchC may help initiate the formation of the nucleocapsid, including interaction with genomic RNA or oligomerization with other C proteins. The C protein is a highly basic protein rich in positively charged amino acid residues (approximately 26 Arg or Lys residues and only 3 negatively charged residues), which is crucial for its binding and interaction with viral RNA. The C protein is a genomic protective agent that can encapsulate viral RNA through its N- and C-terminal basic amino acid clusters to form the viral nucleocapsid. This indicates that the C protein may be a potentially useful target for the development of antiviral therapy [27, 111, 113]. Flavivirus C protein has highly divergent sequences and different domain organizations. However, all C proteins can promote nucleic acid annealing and enhance hammerhead ribozyme-mediated cleavage. The chaperone activity of the flavivirus C protein is very relevant due to its intrinsic disorder; it has the ability to resist heat denaturation, and boiling C protein for 5 min has no effect on its kinetics [114, 115]. Importantly, the C protein may promote profound structural rearrangement of RNA without consuming ATP. C proteins are conserved in *Flaviviridae*, including the *Hepacivirus* (HCV, GBV-B), *Pestivirus* (bovine viral diarrhoea virus, BVDV), and *Flavivirus* genera (WNV). In HCV and GBV-B, the N-terminal region of the C protein is a highly basic and flexible RNA-binding domain, while the C-terminal region is a hydrophobic domain [114]. The central hydrophobic region of the C protein may be related to the ER membrane, which is thought to promote nucleocapsid assembly [45]. In addition, the C protein has variable degrees of tolerance to structural changes and can tolerate extensive deletions in its N-/C-terminus, which indicates that the C protein does not require a defined tertiary structure for its function but relies on basic residues to recruit viral RNA. Due to its structural and functional flexibility, the C protein may be a novel and attractive target for the targeted attenuation of flaviviruses. For example, small-molecule inhibitors of the C protein binding sites may be of interest. Recently, it has been proven that the C proteins of ZIKV and YFV can inhibit small RNAs based on the antiviral response [116].

As a “nonstructural” protein, the C protein precursor anchC plays an important role in flavivirus assembly. Prior cleavage at the anchC dibasic site may affect the cleavage mediated by signalase in the ER lumen, and the sequential cleavage of the anchC sequence is considered to be essential for flavivirus production. The mature C protein can regulate virus replication or change the host cell environment [8, 47, 117]. It plays an important role in virus replication through interactions with various host factors [40], such as B23 [118], Jab1, hnRNP K, and hnRNP A2 [119, 120]. The interaction with organelle membranes of the C protein also plays a vital role in the virus life cycle, but the mechanism by which the interactions affect virus propagation during the virus infection cycle has not been reported. Inhibition of the interaction may severely affect virus replication, assembly and release, thereby reducing virus production. Whether or how the conformation of the C protein changes in this process, which region of the four α-helices (α1 to α4) is involved, and how to specifically label the membranes are all worthy of consideration. Understanding and solving these issues will help us to further study antiviral drugs. The interactions between the C protein and host proteins are conserved in DENV, WNV, and ZIKV [23]. The C protein also plays a crucial role in modulating host cell signalling networks by promoting innate immunity or affecting cell apoptosis, which benefits or impedes the flavivirus replication. The C protein also has favourable antigenicity and can induce host cell-mediated immunity [121, 122] and the humoural immune response, so it can be considered a vaccine target candidate. Drugs that interfere with the formation of the C protein or inhibit its conformational changes during the interactions between the C protein and other proteins will affect the process of genome encapsidation and virus release. Overall, the extraordinary functional flexibility of the C protein makes it an attractive target for flavivirus vaccines and vector engineering design, which is very promising and attractive.

Research on the structure and function of flavivirus C proteins provides us with broad application prospects. The existence of a domain with antibacterial activity in the C protein enhances the importance of viral proteins in the drug development platform and poses new challenges to the coevolution of viruses and bacteria. We expect to make increased efforts in the development of vaccines and drugs.

## Abbreviations

C: Capsid
USUV: Usutu virus
DENV: Dengue virus
JEV: Japanese encephalitis virus
WNV: West Nile virus
ZIKV: Zika virus
TBEV: Tick-borne encephalitis virus
ORF: Open reading frame
UTR: Untranslated region
ER: Endoplasmic reticulum
E: Envelope
NS: Nonstructural
anchC: Anchored C
Ve: Vesicle
VPs: Vesicle packets
COPII: Coat protein complex II
NMR: Nuclear magnetic resonance
TMUV: Tembusu virus
NC: Nucleocapsid
NLSs: Nuclear localization signals
HCV: Hepatitis C virus
DCS-PK: Downstream of the 5′ cyclization sequence pseudoknot
SL6: Stem-loop 6
CPPs: Cell-penetrating peptides
YFV: Yellow fever virus
CTVI: Capsid-targeted antiviral inactivation
TMD: Transmembrane domain
HIS: Internal hydrophobic sequence
LDs: Lipid droplets
VLDL: Very low-density lipoproteins
NMD: Nonsense-mediated mRNA decay
UPF1: Up-frameshift protein 1
hSec3p: Human Sec 3
SG: Stress granule
PI3K: Phosphatidylinositol 3-kinase
CSFV: Classical swine fever virus
EDIII: Envelope domain III
